# Distinct Rates and Transmission Patterns of Major HIV-1 Subtypes among Men who Have Sex with Men in Guangxi, China

**DOI:** 10.3389/fmicb.2023.1339240

**Published:** 2024-01-12

**Authors:** Xianwu Pang, Bo Xie, Qin He, Xing Xie, Jinghua Huang, Kailing Tang, Ningye Fang, Haoming Xie, Jie Ma, Xianmin Ge, Guanghua Lan, Shujia Liang

**Affiliations:** ^1^Guangxi Key Laboratory of Major Infectious Disease Prevention Control and Biosafety Emergency Response, Guangxi Key Laboratory of AIDS Prevention Control and Translation, Guangxi Zhuang Autonomous Region Center for Disease Control and Prevention, Nanning, Guangxi, China; ^2^School of Information and Management, Guangxi Medical University, Nanning, Guangxi, China; ^3^The First Affiliated Hospital of Guangxi Medical University, Guangxi Medical University, Nanning, Guangxi, China

**Keywords:** HIV-1, subtype, diversity, molecular network, MSM

## Abstract

The diversity and transmission patterns of major HIV-1 subtypes among MSM population in Guangxi remains unknown. Understanding the characteristics is crucial for effective intervention strategies. Between 2016 and 2021, we recruited individuals newly diagnosed with HIV-1 from MSM population in Guangxi. HIV-1 pol region was amplified and sequenced, and constructed molecular network, assessed clustering rate, cluster growth rate, spatial clustering, and calculating the basic reproductive number (R0) based on sequences data. We identified 16 prevalent HIV-1 subtypes among Guangxi MSM, with CRF07_BC (53.1%), CRF01_AE (26.23%), and CRF55_01B (12.96%) predominating. In the network, 618 individuals (66.17%) formed 59 clusters. Factors contributing to clustering included age < 30 years (AOR = 1.35), unmarried status (AOR = 1.67), CRF07_BC subtype (AOR = 3.21), and high viral load (AOR = 1.43). CRF07_BC had a higher likelihood of forming larger clusters and having higher degree than CRF01_AE and CRF55_01B. Notably, CRF07_BC has higher cluster growth rate and higher basic reproductive number than CRF01_AE and CRF55_01B. Our findings underscore CRF07_BC as a prominent driver of HIV-1 spread among Guangxi’s MSM population, highlighting the viability of targeted interventions directed at specific subtypes and super clusters to control HIV-1 transmission within this population.

## Introduction

Acquired immune deficiency syndrome (AIDS) remains a formidable global public health challenge, with the escalating HIV infection rates among the Men who have Sex with Men (MSM) population posing a significant concern for China and the global community alike (Huang et al., 2015; Dong et al., 2019). MSM individuals face elevated risks of HIV transmission due to their engagement in irregular partnerships, multiple sexual encounters, unprotected sex, and drug abuse (Chow et al., 2015). The HIV infection rate among MSM population has been reported to be 4.54 times higher than that among the general male population, reflecting the unique vulnerabilities within this demographic (Hessou et al., 2019). Disturbingly, China has observed a rising trend in HIV infection rates among the MSM population over the years, with figures indicating an increase from 0.9% in 2003 to 8.0% in 2015 (SCaWCO, 2015). This escalating infection rates underscores the urgent need to dissect the multifaceted factors driving HIV transmission, particularly within specific regions like Guangxi, China, where MSM play a pivotal role in shaping the epidemic’s trajectory (Pang et al., 2021; Hou et al., 2023).

The HIV-1 virus, characterized by its single plus-stranded RNA structure, exhibits high mutation and recombination rates, contributing to a vast array of genetic diversity (Roberts et al., 1988). This diversity exhibits geographical and demographic nuances (Hemelaar et al., 2011). In China, there exists a multifaceted and diverse spectrum of HIV-1 subtypes. National molecular epidemiological survey have identified more than 11 circulating recombinant forms (CRFs) among HIV-1 infected individuals, with CRF01_AE, CRF07_BC, CRF08_BC, and B subtypes accounting for the majority at 92.8% (He et al., 2012). These subtypes are notebly associated with distinct infectious routes and geographic regions (He et al., 2012; Su et al., 2014; Li et al., 2016). The HIV-1 epidemic in MSM population predominantly features CRF01_AE, B, and CRF07_BC subtypes (Zhang et al., 2015). Across different regions of China, subtype prevalence exhibits variations, such as CRF01_AE being more prevalent in southern provinces, CRF07_BC in eastern provinces, CRF08_BC and C in southwestern provinces, and B in northern provinces (He et al., 2012). CRF55_01B, a newly discovered recombinant subtype, was first identified in Chinese MSM in 2012 (Han et al., 2015), and has been reported in MSM across various provinces in recent years (Beyrer et al., 2012). This diverse distribution of subtypes underscores the intricate interplay among the virus, geography, and infectiou routes.

Guangxi, a southwestern Chinese province bordering Vietnam, has a severe HIV-1 epidemic in China and exhibits complex transmission pattern and multiple CRFs (Ge et al., 2019; Liu et al., 2020; Chen et al., 2021). Despite this, the specific transmission characteristics of HIV-1 in this region remains unclear. The MSM population, concealed due to cultural, social, and familial stigma, constitutes a hidden transmission group. This obscured status, coupled with hidden activities and frequent mobility, presents technical challenges in effectively identifying potential HIV-1 transmission within the MSM population (Tang et al., 2017). Molecular cluster-based transmission network analysis, an innovative methodology, bridges the gap between molecular insights and traditional epidemiological practices by seamlessly integrating social behavioral data (Oster et al., 2018; Ragonnet-Cronin et al., 2018; Jovanović et al., 2019). This integrated approach facilitates a comprehensive understanding of HIV-1 transmission dynamics, particularly among MSM population in Guangxi. By accurately identifying transmission clusters, interventions can be rapidly deployed to interrupt transmission chains and curb further virus spread.

In this study, we first constructed molecular network using pol sequences, and analyzed the characteristics and influencing factors of the molecular network. Furtherly, we predicted the basic reproductive number of major subtypes based on the sequences data and identified the mutation between major subtypes and populations. Finally, the effects of distinct rates and transmission patterns of major HIV-1 subtypes on the prevalence of HIV-1 in MSM population in Guangxi were demonstrated.

## Materials and methods

### Ethics statement

This study was approved by the Ethics Review Board of Guangxi Center for Disease Control and Prevention (Certificate No. GXIRB2016-0047-1). All participants provided their written informed consent to participate in the study, allowing the use of the demographic information and clinical records in future epidemiological studies.

### Study participants and sample collection

Participants were recruited from voluntary counseling and testing centers (VCT) and Non-Governmental Organizations (NGO) in Guangxi from January 2016 to December 2021. These individuals must meet the following requirements: (1) newly diagnosed with HIV-1; (2) had not received ART; (3) age ≥ 18 years old; (4) followed the informed consent of participants. Peripheral blood samples were obtained, and epidemiological data were collected. Plasma was isolated within 12 h of blood collection and stored at −80°C until further analysis.

### RNA extraction and sequencing

The procedures adhered to a previously established protocol. Viral RNA was extracted from plasma using the QIAamp Viral RNA Mini Kit (Qiagen, Hilden, Germany), following manufacturer guidelines. The targeted 1,316 bp fragment within the pol gene (HXB2: 2147–3,462), encompassing protease and the initial 299 reverse transcriptase residues, was amplified through nested polymerase chain reaction (Thermo, United States). Amplified PCR products underwent Sanger sequencing externally (ThermoFisher scientific, ABI3500, United States).

### Sequences processing and HIV-1 subtyping

Firstly, raw sequences were edited using Sequencher v5.1 software (Genecodes, Ann Arbor, MI) and aligned using BioEdit 7.1 software (Ibis Biosciences, Carlsbad, CA, USA). For subtyping, a comprehensive set of 117 reference sequences was utilized, encompassing all Chinese subtypes, which were downloaded from the Los Alamos HIV database, spanning major HIV-1 subtypes, and circulating recombinant forms (CRFs). Lastly, phylogenetic tree was constructed in MEGA 11.0 software by using the neighbor-joining method to identify subtypes.

### Molecular network construction

In the section, we constructed molecular network based on pol sequences. Firstly, the pairwise Tamura-Nei93 (TN93) genetic distance was calculated among aligned sequences using HyPhy software. Sequences exhibiting a genetic distance of ≤1.5% were regarded as potential transmission partners. Therefore, pairwise sequences with genetic distances of less than 1.5% were selected as the matrix for constructing the molecular network. Lastly, molecular network was constructed with the matrix through Cytoscape v3.5.1 software.

### Determination of cluster growth rate

To analyze the factors associated with the cluster growth rate, we calculated each cluster growth rate, which was equal to the ratio of the number of newly diagnosed HIV-1 infected individuals entering the cluster in the past 12 months to the square root of the cluster size (Wertheim et al., 2018). The growth rate of non-clustered individual was 0. According to the distribution of cluster growth rate, clusters were categorized as high-growth cluster (top 25%), medium-growth cluster (25–50%), and low-growth cluster (bottom 50%).

### Determination of cluster type

We classify the types of clusters according to their formation time: (1) Growing cluster: comprising ≥3 individuals diagnosed between 2016 and 2018, and ≥ 1 diagnosis in 2021; (2) Emerging cluster: containing diagnoses solely from 2019 to 2021; (3) Stable cluster: consisting only of pre-2018 infections; and (4) other: remaining clusters.

### The basic reproductive number calculation

Calculating the basic reproductive number from HIV-1 sequence data according the previous study (Stadler et al., 2012). Firstly, the pol sequences were imported into Fasttree software by employing the Maximum Likelihood (ML) model for producting Newick file. Secondly, the Newick file was subjected to correlation analysis using TempEst software, requiring a correlation coefficient above 0.3. Thirdly, the Bayesian model parameters were setted using the Birth Death Skyline Serial model in BEAUTi software, and subsequently, BEAST v2.7.4 software and TreeAnnotator software generated the Maximum Clade Credibility (MCC) trees. Lastly, the “bdskytools” package (sourced from GitHub) was employed for visualizing the dynamic R0 in R software.

### HIV-1 Lag-avidity enzyme immunoassay (EIA)

HIV-1 LAg-Avidity enzyme immunoassay was used to identified recent infection and long-term infection. These individuals who had been treated with antiretroviral therapy (ART) and CD4+ T cell<200 was excluded for testing. The testing was performed according to the manufacturer’s instructions (Sedia, Portland, OR, USA). Test specimens were initially run as single samples. If the normalized OD (ODn) was>2.0, the specimen was classifed as a long-term infection. Specimens with an ODn < 2.0 were tested again in triplicate to confirm the obtained values. In confrmatory testing, specimens with an ODn < 1.5 were classifed as recent infections.

### Statistical analysis

Demographic characteristics of the study participants were summarized using frequencies and percentages. For non-normally distributed data, the median was employed. To assess influencing factors for clustering and cluster growth rate, a multifactor logistic regression model was applied. The crude odds ratios (COR) adjusted odds ratios (AOR), and 95% confidence intervals (CI) were calculated. The chi-square test examined factors associated with cluster size and degree. A bilateral *value of p* test was performed, and significance was established at *p* < 0.05. Mapping and visualization were accomplished using GraphPad Prism 9 and R Studio software. IBM SPSS 26 software was utilized for statistical analysis.

## Results

### Characteristics of HIV-1 molecular networks

A total of 934 HIV-1pol sequences were acquired, and 618 (66.17%) forming 59 distinct molecular clusters. Notably, a progressive growth of molecular networks from 2016 to 2021 emerged, primarily dominated by several large clusters. The number of clusters increased from 11 in 2016 to 51 in 2021. A notable cluster expanded from 27 individuals in 2016 to 340 individuals in 2021, and degrees range from 1 to 183 (Supplementary Figure S1).

**Figure 1:**
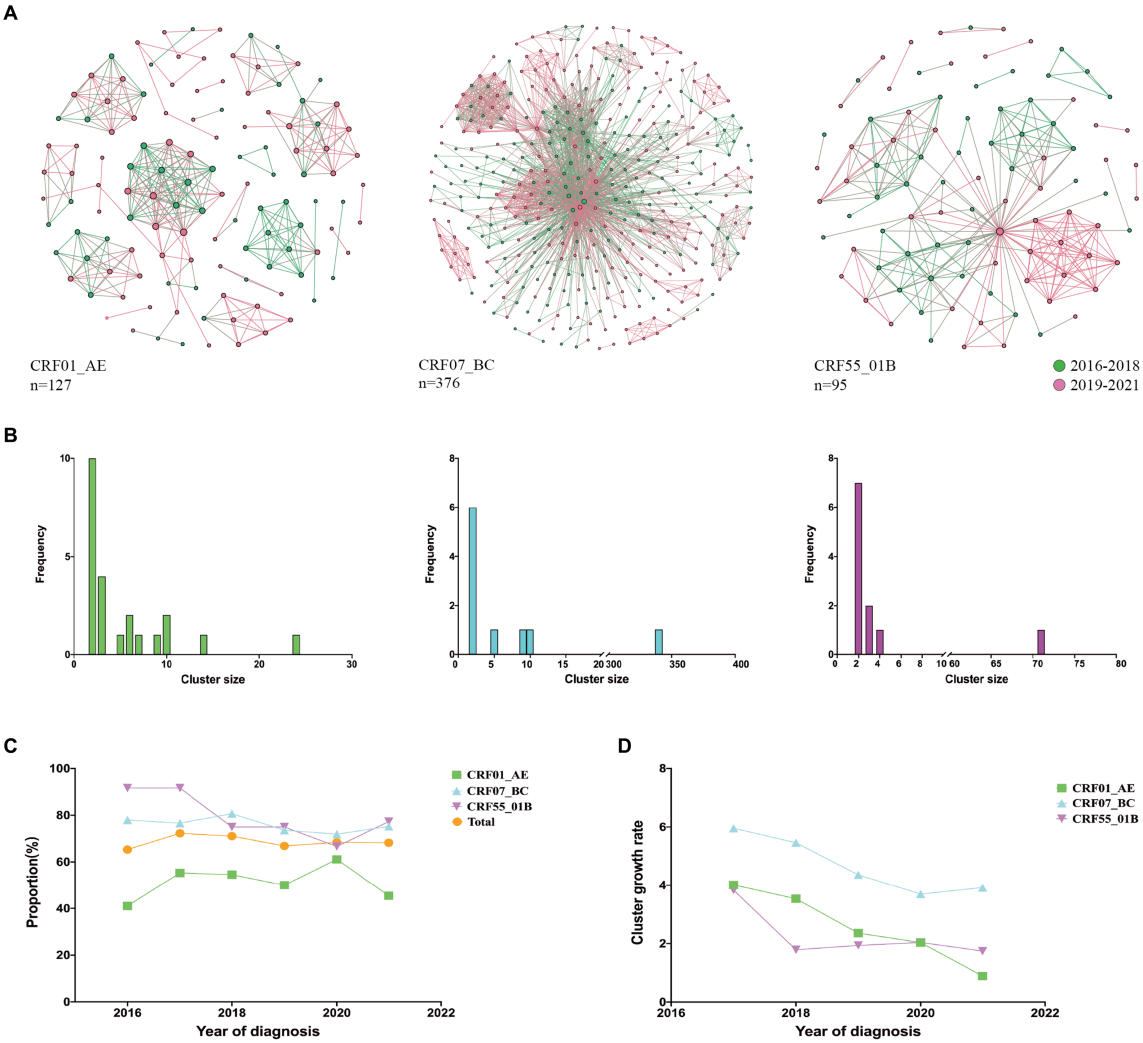
Molecular network characteristics of major HIV-1 subtypes. **(A)**: Transmission networks formed by three major subtypes; **(B)**: Distribution of cluster size of three major subtypes; **(C)**: Clustering rates of three major subtypes from 2016 to 2021; **(D)**: Cluster growth index of three major subtypes from 2016 to 2021.

We compared the characteristics of molecular networks of three major subtypes CRF01_AE, CRF07_BC, and CRF55_01B (Figure 1A). Notably, CRF01_AE exhibited the highest proportion of small clusters (nodes <5, 78.26%), followed by CRF55_01B (63.64%), while CRF07_BC showcased the greatest share of large clusters (nodes >10, 50%) (Figure 1B). From 2016 to 2021, the highest clustering rates were CRF55_01B, but the clustering rate decreased from 91.67 to 77.27%; while the clustering rate of CRF07_BC (mean clustering rate: 75.81%) and CRF01_AE (mean clustering rate: 51.84%) kept stable (Figure 1C). Remarkably, CRF07_BC displaying the highest cluster growth rate (Figure 1D).

**Figure 2:**
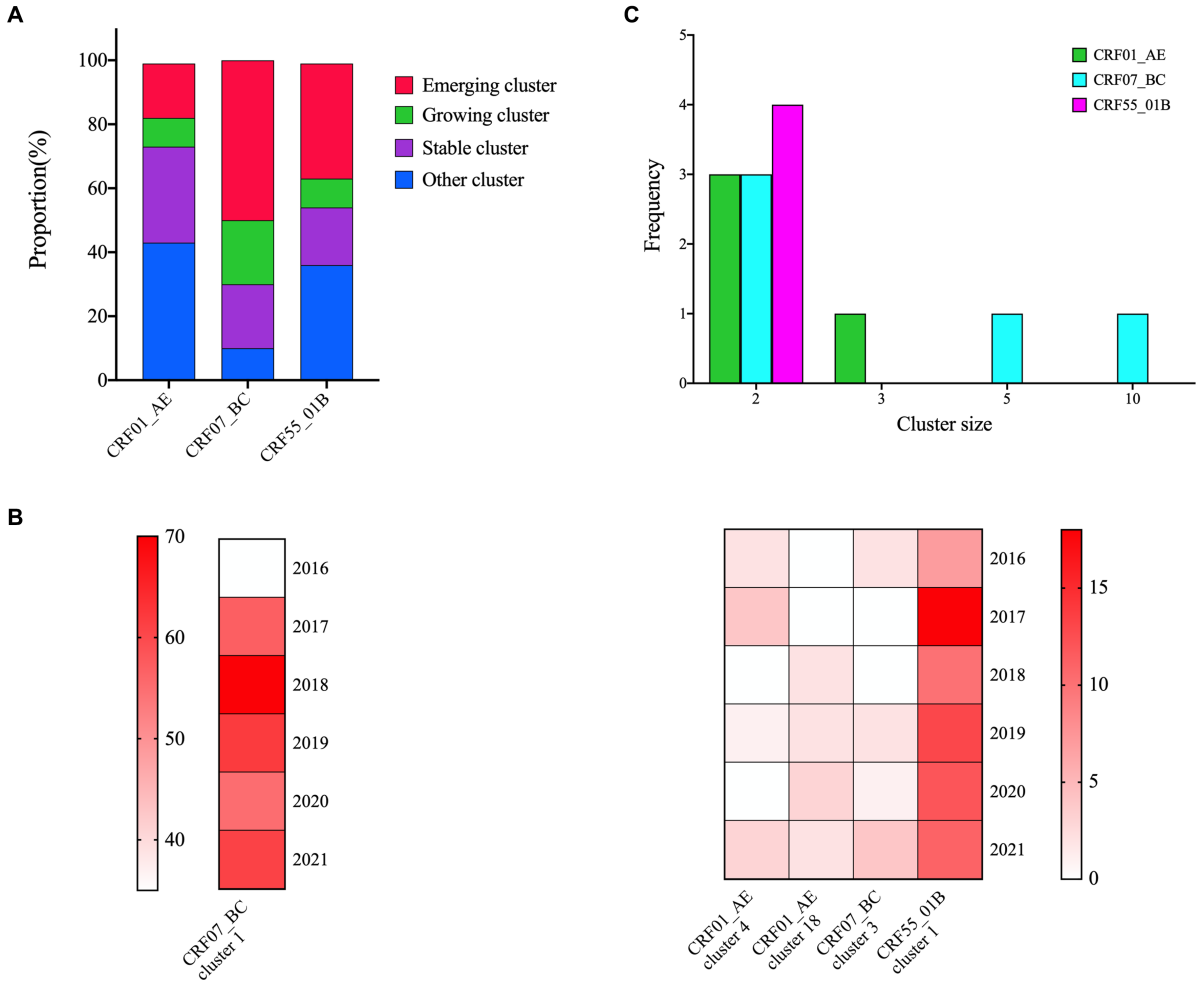
Cluster types of major HIV-1 subtypes. **(A)**: The proportion of cluster types in the three networks; **(B)**: 5 growing clusters identified in this study; the shade of each color block represents the number of new sequences added each year; **(C)**: The distribution of the emerging clusters of three subtypes.

In the cluster type analysis results, CRF07_BC exhibited the highest proportion of emerging and growth clusters, followed by CRF55_01B, while CRF01_AE displayed the lowest proportion (Figure 2A). Within five identified growth clusters, which were CRF07_BC cluster 1 and 3, CRF01_AE cluster 4 and 18, and CRF55_01B cluster 1. CRF07_BC cluster 1 was the largest cluster and comprised 340 individuals, increased from 35 individuals in 2016 to 340 in 2021. The second cluster was CRF55_01B cluster 1, containing 71 individuals, increased from 7 individuals in 2016 to 71 in 2021 (Figure 2B). 13 emerging clusters were identified, 5 clusters belong to CRF07_BC (cluster size: 10, 5 and 2, respectively), 4 clusters were CRF55_01B (cluster size: 2) and 4 clusters for CRF01_AE (cluster size: 2 and 3) (Figure 2C).

### The influencing factors of HIV-1 molecular networks

Multivariate logistic regression analysis unveiled significant risk factors impacting HIV clustering in MSM population. Population younger than 30 years old (aOR = 1.35) were more likely to form clusters than the age (30–49). Being unmarried (aOR = 1.67) elevated clustering risk compared to being married. People infected with CRF07_BC (aOR = 3.21) and CRF55_01B (aOR = 3.63) were more likely to form clusters than other subtypes (aOR = 0.49). High viral load (aOR = 1.43) was also associated with an increased likelihood of clustering. The above results are presented in Table 1.

**Table 1:** Analysis of factors associated with HIV-1 clustering in MSM population.

Variables	Number (%)		OR (95% CI)	
	Total (934)	Within networks	Unadjusted	Adjusted
Age				
< 30	601 (64.35)	451 (72.98)	ref	ref
30–49	277 (29.66)	140 (22.65)	0.66 (0.49–0.89)**	0.74 (0.42–1.29)*
≥ 50	56 (6)	27 (4.37)	0.63 (0.34–1.15)	0.98 (0.33–2.95)
Marital status				
Married	134 (14.35)	41 (6.63)	ref	ref
Unmarried	755 (80.84)	552 (89.32)	1.90 (1.28–2.83)***	1.67 (1.00–2.81)*
Divorced/widowed	45 (4.82)	25 (4.05)	1.09 (0.54–2.17)	1.11 (0.54–2.26)
Educational level				
College and above	583 (62.42)	396 (64.08)	ref	ref
High school or technical school	211 (22.59)	142 (22.98)	0.97 (0.69–1.36)	1.02 (0.71–1.45)
Junior high school and below	140 (14.99)	80 (12.94)	0.62 (0.42–0.93)*	0.72 (0.46–1.13)
Ethnicity				
Han	553 (59.21)	368 (59.55)	ref	
Zhuang	336 (35.97)	220 (35.6)	0.95 (0.72–1.27)	
Other	45 (4.82)	30 (4.85)	1.01 (0.53–1.92)	
Subtype				
CRF01_AE	245 (26.23)	123 (19.9)	ref	ref
CRF07_BC	496 (53.1)	376 (60.84)	3.11 (2.25–4.30)***	3.21 (2.30–4.49)***
CRF55_01B	121 (12.96)	95 (15.37)	3.62 (2.20–5.98)***	3.63 (2.18–6.04)***
Other	72 (7.71)	24 (3.88)	0.50 (0.29–0.86)*	0.49 (0.28–0.87)*
Occupation				
Unemployed	240 (25.7)	160 (25.89)	ref	
Individual business	139 (14.88)	87 (14.08)	0.84 (0.54–1.29)	
Student	171 (18.31)	117 (18.93)	1.08 (0.71–1.65)	
Clerk	209 (22.38)	141 (22.82)	1.04 (0.70–1.54)	
Workers	96 (10.28)	61 (9.87)	0.87 (0.53–1.43)	
Services	79 (8.46)	52 (8.41)	0.96 (0.56–1.65)	
Infection of other STDs				
No	769 (82.33)	515 (83.33)	ref	ref
Yes	165 (17.67)	103 (16.67)	0.82 (0.58–1.16)	0.88 (0.61–1.25)
Number of sexual partners				
1–4	326 (34.9)	214 (34.63)	ref	ref
5–9	261 (27.94)	171 (27.67)	0.99 (0.71–1.40)	0.99 (0.70–1.40)
≥10	347 (37.15)	233 (37.7)	1.07 (0.78–1.47)	1.10 (0.79–1.53)
Infection time				
Long-term	533 (57.07)	346 (55.99)	ref	
Recent	313 (33.51)	212 (34.3)	1.13 (0.84–1.53)	
Unknow	88 (9.42)	60 (9.71)	1.10 (0.82–1.49)	
Pre-treatment CD4 T cell counts, cells/mm				
< 200	132 (14.13)	87 (14.08)	ref	ref
200–350	303 (32.44)	207 (33.5)	1.12 (0.72–1.72)	1.10 (0.71–1.72)
>350	499 (53.43)	324 (52.43)	0.96 (0.64–1.44)	0.94 (0.62–1.43)
Viral load (Log10)	934 (100)	618 (100)	1.33 (1.02–1.73)*	1.43 (1.08–1.90)*
Region				
Xixiangtang	282 (30.19)	201 (32.52)	ref	ref
Qingxiu	234 (25.05)	154 (24.92)	0.78 (0.53–1.13)	0.74 (0.50–1.08)
Jiangnan	132 (14.13)	84 (13.59)	0.71 (0.46–1.09)	0.72 (0.46–1.12)
Xingning	81 (8.67)	51 (8.25)	0.69 (0.41–1.15)	0.71 (0.41–1.21)
Liangqing	44 (4.71)	30 (4.85)	0.86 (0.44–1.71)	1.03 (0.51–2.08)
Other	161 (17.24)	98 (15.86)	0.63 (0.42–0.94)*	0.66 (0.44–1.01)
Year of diagnosis				
2016	109 (11.67)	67 (10.84)	ref	
2017	158 (16.92)	109 (17.64)	1.39 (0.84–2.33)	
2018	174 (18.63)	118 (19.09)	1.32 (0.80–2.18)	
2019	173 (18.52)	113 (18.28)	1.18 (0.72–1.94)	
2020	159 (17.02)	106 (17.15)	1.25 (0.76–2.08)	
2021	161 (17.24)	105 (16.99)	1.18 (0.71–1.95)	

*p < 0.05, **p < 0.01, ***p < 0.001.

Chi-square test results showed that the distribution of cluster size was statistically different in age (*p* < 0.001), marital status (*p* = 0.003), education level (*p* = 0.013) and subtype (*p* < 0.001) (Supplementary Table S1). Age under 30, unmarried individuals, those with a college degree or higher, and CRF07_BC subtype exhibited a heightened tendency to form larger cluster. Chi-square testing underscored significant associations between degree and age (*p* = 0.003), marital status (*p* < 0.001), and CRF07_BC subtype (*p* < 0.001) (Supplementary Table S2). Individuals, age under 30, unmarried, and those infected with the CRF07_BC subtype demonstrated an elevated propensity for higher degree.

### The cluster growth rate of major HIV-1 subtypes

The cluster growth rates spanned 0 to 5.94, with a median of 1.0 (IQR 0.00–3.31), presenting a right-skewed distribution (Supplementary Figure S2). The thresholds for high-growth clusters (n = 207), medium-growth clusters (n = 236) and low-growth clusters (n = 447) were 3.6, 1.33 and 0, respectively. Chi-square test results showed significant differences in HIV-1 subtypes, long-term infection, pre-treatment viral load, and diagnosis year (*p* < 0.001). Of note, 100% of the high-growth clusters belonged to the CRF07_BC subtype, while no such subtype was detected in the low-growth and medium-growth clusters. In contrast, 80.2 and 43.4% of the CRF01_AE and CRF55_01B subtypes belong to low-growth clusters, and 19.8 and 56.6% belong to medium-growth clusters, respectively (Supplementary Table S3).

To further study the factors related to the cluster growth rate, we categorized cluster growth rate into two groups and conducted multivariate logistic regression analysis. When the growth rates were categorized as low growth rate (<1.33) and high growth rate (≥1.33), age < 30, CRF07_BC, and CRF55_01B were risk factors for high growth rate. When growth rates were divided into low growth rate (<3.6) and high growth rate (≥3.6), long-term infection and CRF07_BC were risk factors for high growth rate (Table 2).

**Table 2:** Analysis of factors associated with the cluster growth rate.

Variables	Cluster growth≥1.33	*p* value	Cluster growth≥3.6	*p* value
	OR (95% CI)		OR (95% CI)	
Age				
< 30	ref		ref	
30–49	0.69 (0.51–0.92)	0.013	0.83 (0.58–1.19)	0.311
≥ 50	0.75 (0.40–1.38)	0.350	1.01 (0.49–2.06)	0.982
Marital status				
Married	ref		ref	
Unmarried	1.49 (0.98–2.25)	0.062	1.25 (0.75–2.07)	0.397
Divorced/widowed	1.23 (0.60–2.52)	0.565	0.90 (0.37–2.23)	0.826
Educational level				
College and above	ref		ref	
High school or technical school	1.15 (0.83–1.60)	0.389	1.33 (0.92–1.93)	0.134
Junior high school and below	0.71 (0.48–1.04)	0.076	0.89 (0.56–1.42)	0.631
Ethnicity				
Han	ref		ref	
Zhuang	0.90 (0.68–1.19)	0.464	0.84 (0.60–1.17)	0.292
Other	1.12 (0.60–2.10)	0.713	1.07 (0.52–2.18)	0.855
Subtype				
CRF01_AE	ref			
CRF07_BC	6.82 (4.71–9.89)	<0.001	ref	
CRF55_01B	5.02 (3.09–8.14)	<0.001	0.27 (0.16–0.46)	<0.001
Other	0.43 (0.19–1.01)	0.052		
Occupation				
Unemployed	ref		ref	
Individual business	1.10 (0.72–1.69)	0.659	1.40 (0.86–2.29)	0.180
Student	1.13 (0.76–1.69)	0.556	1.48 (0.93–2.34)	0.099
Clerk	1.11 (0.76–1.63)	0.584	0.87 (0.54–1.39)	0.555
Workers	1.03 (0.64–1.67)	0.898	0.76 (0.41–1.43)	0.396
Services	1.34 (0.79–2.29)	0.281	1.44 (0.79–2.64)	0.238
Infection of other STDs				
No	ref		ref	
Yes	0.80 (0.56–1.13)	0.207	0.79 (0.52–1.22)	0.291
Number of sexual partners				
1–4	ref		ref	
5–9	1.08 (0.77–1.51)	0.669	0.73 (0.49–1.09)	0.121
≥10	1.20 (0.88–1.63)	0.252	0.84 (0.58–1.20)	0.333
Infection time				
Long-term	ref		ref	
Recent	1.05 (0.79–1.40)	0.743	0.53 (0.37–0.77)	<0.001
Unknown	1.03 (0.65–1.63)	0.904	1.21 (0.73–1.99)	0.466
Pre-treatment CD4 T cell counts, cells/mm				
<200	ref		ref	
200–350	1.47 (0.97–2.23)	0.073	1.71 (0.98–2.99)	0.058
>350	1.15 (0.77–1.70)	0.502	1.89 (1.11–3.21)	0.019
Viral load (Log10)				
	1.12 (0.88–1.43)	0.360	0.85 (0.59–1.23)	0.392
Region				
Xixiangtang	ref		ref	
Qingxiu	0.86 (0.60–1.23)	0.402	0.96 (0.64–1.46)	0.855
Jiangnan	0.69 (0.45–1.05)	0.081	0.83 (0.50–1.38)	0.483
Xingning	0.61 (0.36–1.01)	0.056	0.76 (0.41–1.42)	0.391
Liangqing	0.79 (0.42–1.50)	0.470	1.16 (0.57–2.38)	0.688
Other	0.56 (0.37–0.83)	0.004	0.91 (0.57–1.45)	0.680
Year of diagnosis				
2017	ref		ref	
2018	1.06 (0.67–1.66)	0.812	0.75 (0.48–1.15)	0.185
2019	0.57 (0.37–0.90)	0.014	0.62 (0.40–0.96)	0.032
2020	0.52 (0.33–0.82)	0.005	-	-
2021	0.59 (0.38–0.94)	0.026		

### Spatial clustering and migration events of major HIV-1 subtype

**Figure 3:**
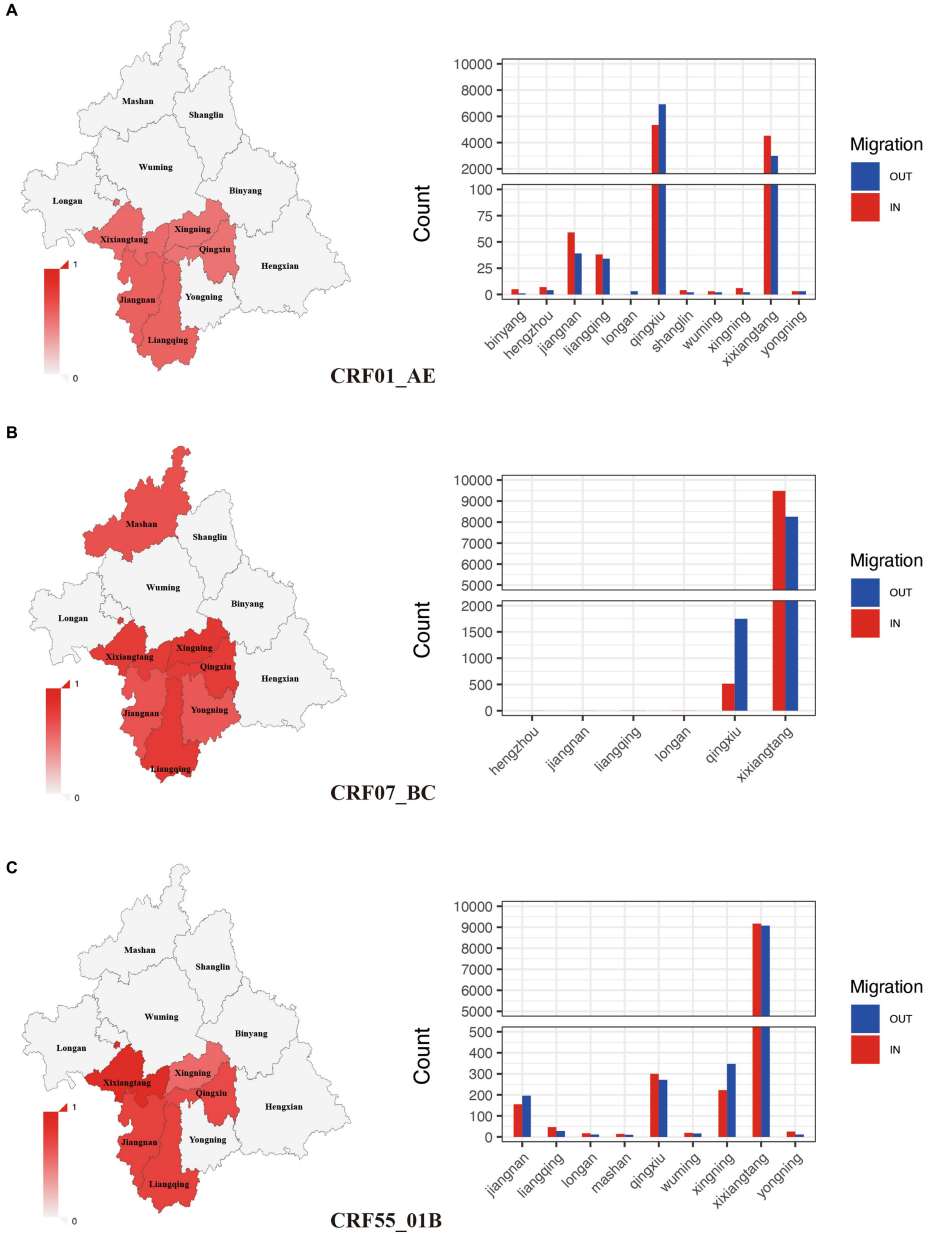
Spatial clustering and migration events of major subtypes. **(A)**: Geographic distribution of clustering rate and migration frequency of CRF01_AE; **(B)**: Geographic distribution of clustering rate and migration frequency of CRF07_BC; **(C)**: Geographic distribution of clustering rate and migration frequency of CRF55_01B.

Among all subtypes, CRF07_BC (75.81%) and CRF55_01B (78.51%) had the highest clustering rate, followed by CRF01_AE (50.2%). These subtypes with high clustering rate were mainly concentrated in Xixiangtang, Qingxiu, and Jiangnan. Within the CRF01_AE subtype, the region with the highest input and output events were Qingxiu and Xixiangtang. In these events, Qingxiu exhibited a net output, while Xixiangtang displayed a net input (Figure 3A). In contrast, within the CRF07_BC subtype, the highest input and output events were observed in Xixiangtang, followed by Qingxiu. In this subtype, Qingxiu acted as the net output area, while Xixiangtang served as the net input location (Figure 3B). For the CRF55_01B subtype, the region with the highest input and output events were also Xixiangtang and Qingxiu, respectively (Figure 3C). It shows that HIV-1 transmission in the region is clustered. Spatial distribution revealed strong correlation between Qingxiu and Xixiangtang Districts (Supplementary Figure S3A). Among the CRF01_AE and CRF07_BC subtypes, the correlation between Qingxiu District and Xixiangtang District was the strongest (Supplementary Figures S3B,C). CRF55_01B subtype exhibited strongest within Xixiangtang District (Supplementary Figure S3D). These findings highlight distinct spatial distribution and transmission in Nanning, the capital of Guangxi.

### The basic reproductive number analysis of major HIV-1 subtypes

**Figure 4:**
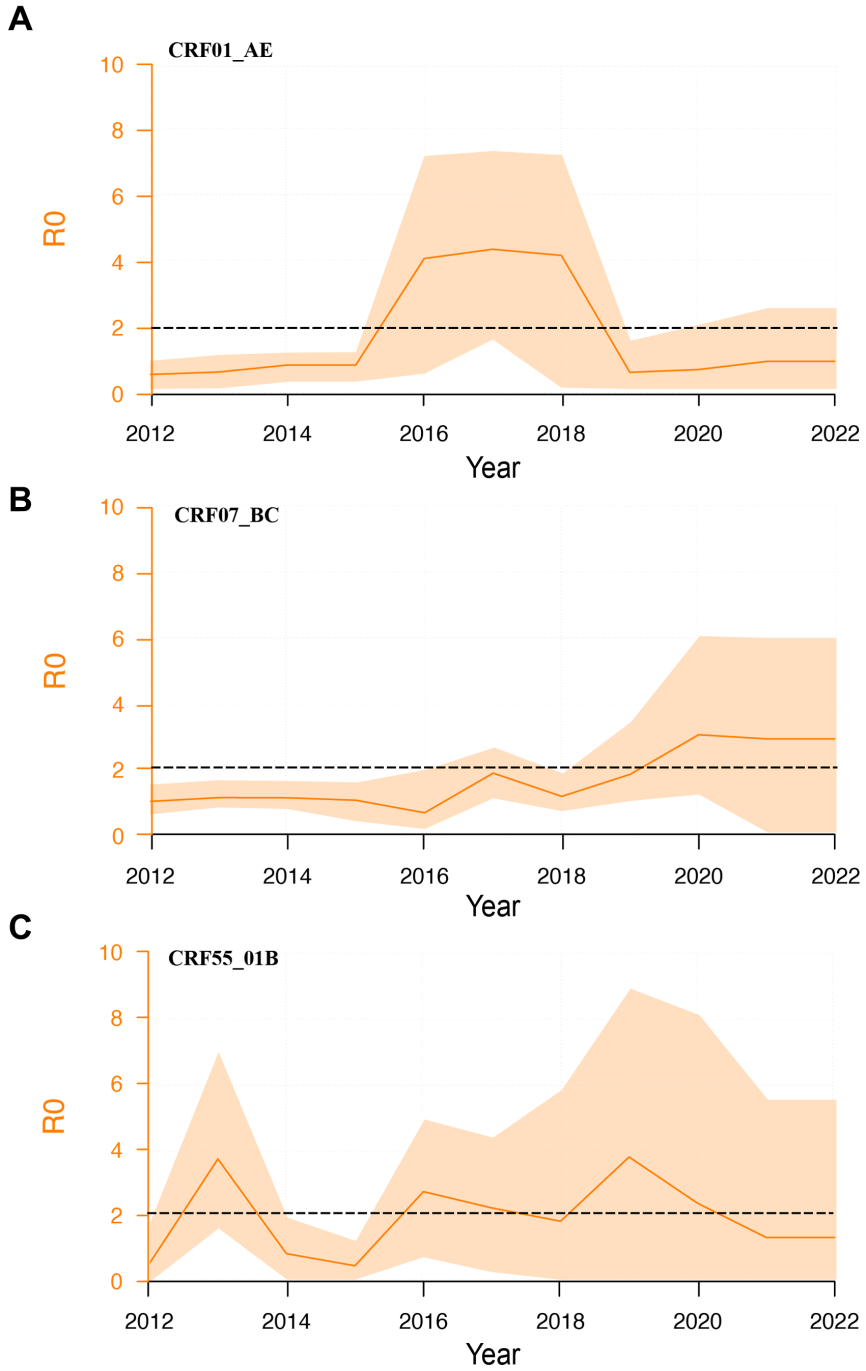
Dynamic R0 of major HIV-1 subtypes. **(A)**: Dynamic R0 of CRF01_01AE subtype; **(B)**: Dynamic R0 of CRF07_BC subtype; **(C)**: Dynamic R0 of CRF55_01B subtype.

To explore the second-generation transmission ability of different subtypes, we calculated their dynamic R0 values. CRF01_AE subtype exhibited a brief surge from 2016 to 2018, stabilizing near 1 thereafter (Figure 4A). CRF55_01B subtypes demonstrated relatively constant R0 values between 1 and 2 (Figure 4C). Contrastingly, CRF07_BC subtype showed an increasing trend, and R0 was higher than 2 (Figure 4B). We further calculated the average R0 values for each subtype between 2016 and 2021.

## Discussion

In this study, we delved into the complex dynamics of HIV-1 transmission among the MSM population in Guangxi, China. The multifaceted insights gained from analyzing molecular networks, the effects of major subtype on clustering, growth rate, spatial distribution, and the basic reproductive number to our understanding of the complex landscape of HIV-1 transmission. These findings have important implications for guiding targeted interventions and strategies to effectively curb the transmission of the virus in MSM populations.

The molecular network analysis uncovered the presence of distinct transmission clusters and the emergence of super clusters, which offer a nuanced view of the transmission dynamics. Mehta et al. (Mehta et al., 2015) constructed molecular networks collected from six states in the United States between 2010 and 2012, monitored the change trend of these networks between 2013 and 2017, and identified the relationship between HIV infection events in rapidly growing molecular clusters. Campbell et al. (Campbell et al., 2017) investigated the large-scale HIV infection outbreak in a county in Indiana, USA in 2015 by constructing molecular networks and found that many groups with HIV negative were closely related to HIV networks. The results indicate that molecular network analysis can be used for targeted follow-up and precise intervention for HIV prevention and control, and to find more people who have social network relationships with people living with HIV and undiagnosed infected people. The prevalence of different clusters and the emergence of large clusters among MSM population in Guangxi indicate a complex interplay of factors affecting the dynamics of HIV-1 transmission. The high proportion of young, unmarried, and highly educated individuals in these groups highlights the vulnerability of population to HIV-1 transmission. These observations are consistent with previous research and highlight the importance of targeted interventions that address the key populations, such as young gay men (Pang et al., 2021). The results of molecular network are of great significance in guiding the accurate prevention and control of HIV. We will take measures to intervene according to the key nodes in the network, prevent secondary transmission, and trace the source through the key nodes to find undiscovered HIV infected people.

The prevalence of HIV-1 subtype is related to geographic region and route of transmission (Su et al., 2014; Li et al., 2016). Spatiotemporal transmission analysis revealed important insights into the geographic transmission of HIV-1. Distribution of HIV-1 positive individuals in specific regions, as well as different clustering rates and subtype distributions, highlight multiple factors influencing the transmission of HIV-1. Strong correlations between certain regions and subtypes suggest potential transmission hotspots. This information is essential for designing geographically targeted interventions for changes in transmission intensity. The main subtype circulating among MSM in Guangxi was CRF07_BC, follow by CRF01_AE and CRF55_01B, which is different from other provinces of China. For instance, CRF01_AE (43.3%) was the major genotype, followed by CRF07_BC (31.3%), and CRF67_01B (7.2%) (Yin et al., 2021). In Tianjin, CRF01_AE (56.9%) was the major genotype, follow by CRF07_BC (27.8%), B (7.3%), CRF55_01B (4.1%) (Zheng et al., 2020). In Kuming, adjacent to Guangxi, the main subtype was CRF01_AE (64.9%), followed by CRF07_BC (25.2%), and the recombinant forms (URFs, 5.3%) (Chen et al., 2014). The different clustering patterns observed in these subtypes further emphasize the need for tailored prevention strategies. The dominance of CRF07_BC in high growth cluster suggests that CRF07_BC may be a high transmission risk factor. Even if CRF01_AE and CRF55_01B were in low-growth clusters, these individuals infected with CRF01_AE and CRF55_01B in clusters may have an impact on HIV-1 transmission before treatment and intervention was initiated. These insights provide valuable information for designing targeted interventions to effectively control and prevent HIV-1 transmission. At the same time, the basic regeneration number analysis showed that the mean R0 of CRF07_BC was higher than that of CRF01_AE and CRF55_01B, indicating that CRF07_BC was more likely to transmission in MSM population in Guangxi than other subtypes, so it is necessary to strengthen the monitoring of this subtype. The molecular mechanism of CRF07_BC’s susceptibility to transmission in MSM population remains to be further explored. It has been reported that individuals infected with CRF07_BC exhibit a lower viral load and slower rate of disease progression compared to individuals infected with HIV-1 subtype B or CRF01_AE (Cheng et al., 2022). Compared with patients infected with HIV-1 subtype B or CRF01_AE, the time from HIV-1 diagnosis to the development of immunodeficiency was significantly longer in those infected with CRF07_BC (Lin et al., 2013; Huang et al., 2014; Jiang et al., 2016). CRF07_BC was first found among IDUs in Guangxi in 2002 (Mcclutchan et al., 2002), and gradually formed a large-scale epidemic in the area in recent years. The above results proved that CRF07_BC was spreading rapidly among MSM population in Guangxi and replaced CRF01_AE as the dominant subtype. In the heterosexual population in Guangxi, CRF01_AE was still the main subtype (Pang et al., 2021). At the same time, we should also be vigilant that CRF55_01B’s cluster growth rate and R0 are second only to CRF07_BC, but its clustering rate is the highest. CRF55_01B was first identifed in MSM population in Chinese in 2012 (Han et al., 2013). In our study published in 2021 (Pang et al., 2021), the proportion of CRF55_01B was 11.3% by 2018 but increased to 15.37% by 2021. Therefore, only by understanding the transmission characteristics of various subtypes in different populations can we formulate corresponding public health policies.

It is important to acknowledge the limitations of our study. While molecular network analysis provides valuable insights, it is based on samples we collected and may not include all infected individuals. In addition, due to social discrimination, some infected individuals did not admit that they belong to the MSM, or did not take the initiative to test, which may lead to the loss of some key infected individuals.

## Conclusion

In conclusion, our results showed that CRF07_BC was more likely to form large clusters and have high degree than other subtypes, and its cluster growth rate and basic reproductive number were greater than those of other subtypes, which underscore CRF07_BC as a prominent driver of HIV-1 spread among MSM population in Guangxi. The insights gained from this study have important implications for designing targeted interventions to effectively control and prevent the transmission of HIV-1 in key population. Future research could further explore the complex interplay between viral genetics, host factors, and behavioral patterns to develop more precise HIV/AIDS prevention and control strategies.

## Data availability statement

The datasets presented in this study can be found in online repositories. The names of the repository/repositories and accession number(s) can be found here: NCBI - MW294209 - MW295352, G1135-G1807.

## Ethics statement

The studies involving humans were approved by Ethics Review Board of Guangxi Center for Disease Control and Prevention. The studies were conducted in accordance with the local legislation and institutional requirements. The human samples used in this study were acquired from primarily isolated as part of your previous study for which ethical approval was obtained. Written informed consent for participation was not required from the participants or the participants' legal guardians/next of kin in accordance with the national legislation and institutional requirements.

## Author contributions

XP: Conceptualization, Data curation, Formal Analysis, Investigation, Methodology, Resources, Software, Validation, Visualization, Writing – original draft, Writing – review & editing. BX: Data curation, Software, Visualization, Writing – review & editing. QH: Data curation, Methodology, Resources, Writing – review & editing. XX: Data curation, Software, Visualization, Writing – review & editing. JH: Data curation, Software, Visualization, Writing – review & editing. KT: Data curation, Methodology, Resources, Writing – review & editing. NF: Data curation, Methodology, Writing – review & editing. HX: Methodology, Resources, Writing – review & editing. JM: Data curation, Resources, Writing – review & editing. LG: Conceptualization, Funding acquisition, Project administration, Writing – review & editing. SL: Conceptualization, Funding acquisition, Project administration, Writing – review & editing. XG: Funding acquisition, Writing – review & editing.
