# Supplementary material for: Distinct Rates and Transmission Patterns of Major HIV-1 Subtypes among Men who Have Sex with Men in Guangxi, China
Source: Front Microbiol. 2024 Jan 12;14:1339240. doi: 10.3389/fmicb.2023.1339240 (PMC10822680; doi:10.3389/fmicb.2023.1339240)
Supplement: Supplementary file 1 [file Data_Sheet_1.docx]

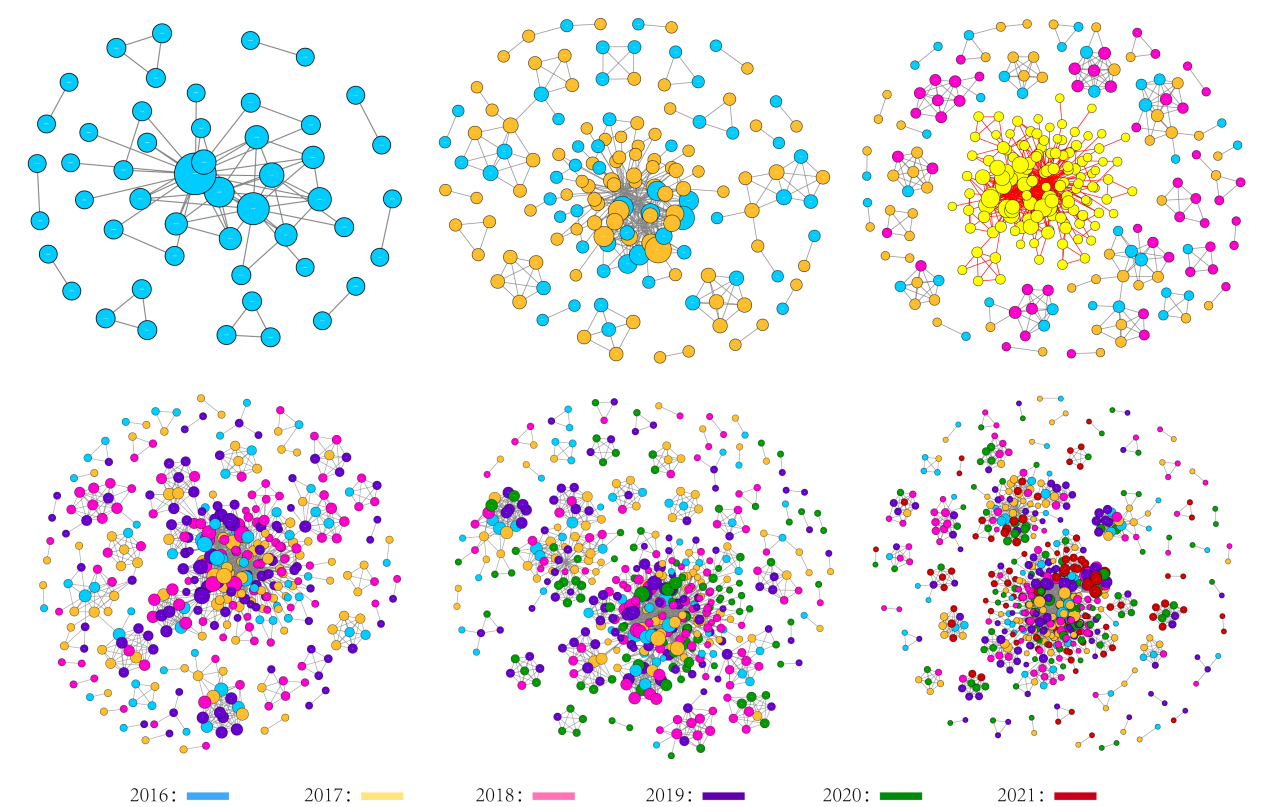


**Figure S1. Changes of HIV-1 molecular network from 2016 to 2021.** Colors represent different year. Nodes indicate HIV patients or sequences. The size of a node is proportional to its degree. Edges (i.e., links) represent genetic linkage.


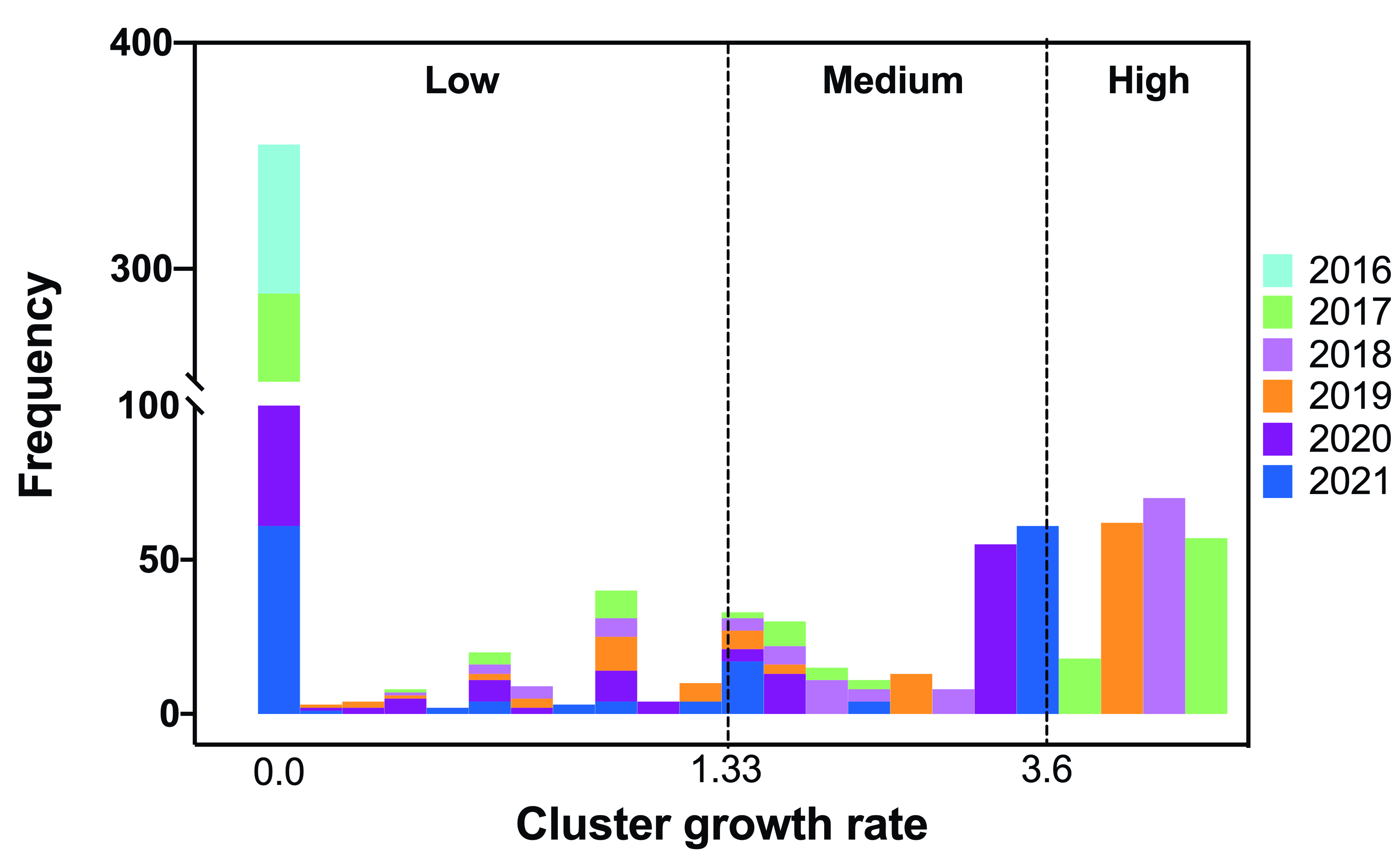


**Figure S2. The distribution of cluster growth rate.** The cluster growth rate was divided into low (<1.33), medium (1.33-3.6), high (>3.6). Colors represent different year.


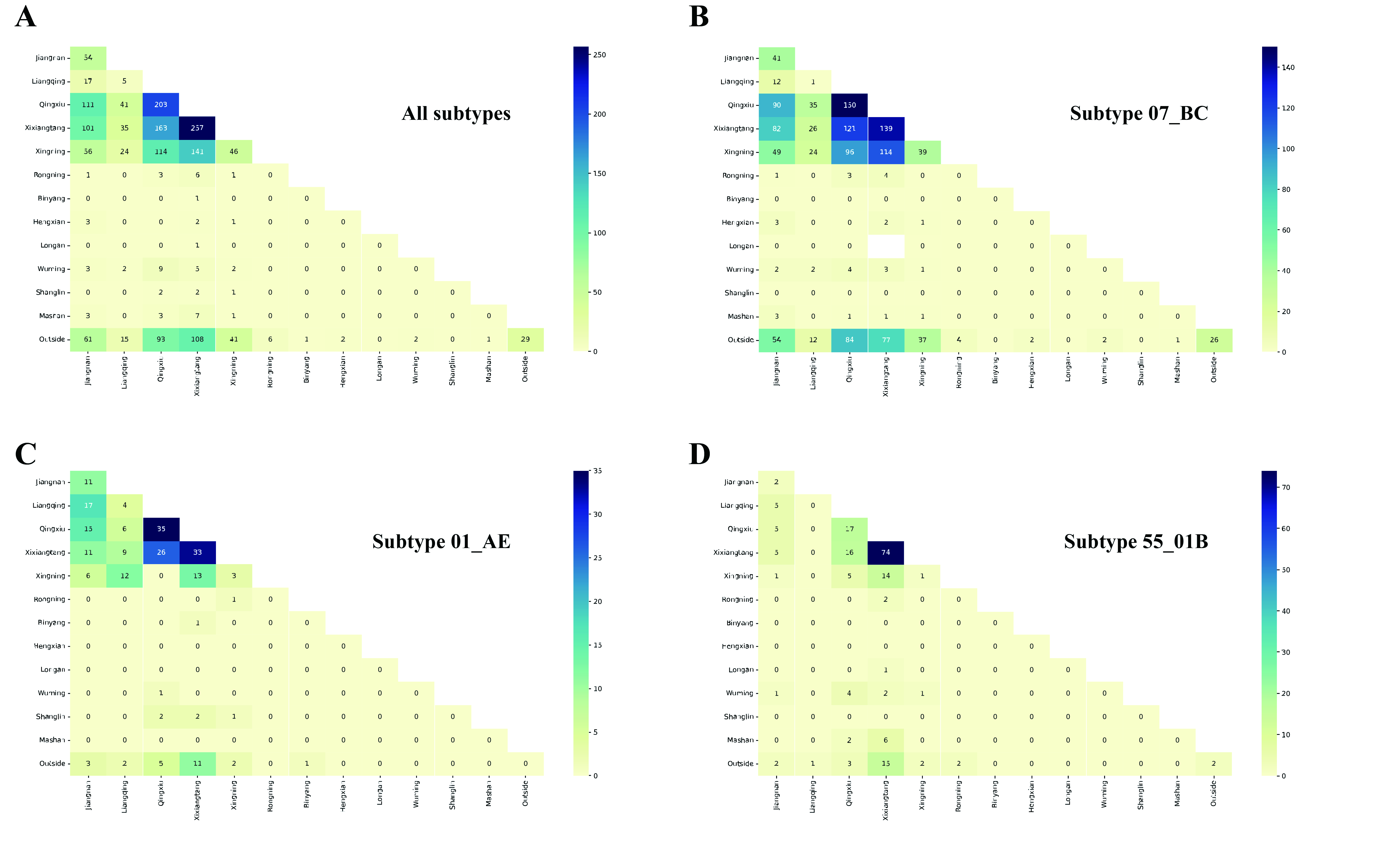


**Figure S3. Strength matrix analysis of HIV-1 transmission between regions.** Grid values indicate horizontal and vertical correlation intensity; higher values denote stronger inter-region correlation. A: Strength matrix analysis of all subtypes between regions; B: Strength matrix analysis of CRF07_BC between regions; C: Strength matrix analysis of CRF01_AE between regions; D: Strength matrix analysis of CRF55_01B between regions.

Table S1 Characteristic analysis of different cluster size.

| Variables | Cluster size（%） | | | | | *χ ^2^* | *p* |
| --- | --- | --- | --- | --- | --- | --- | --- |
|  | unclustered | 2-4 | 5-10 | 11-30 | >30 |  |  |
| Age |  |  |  |  |  | 31.09 | <0.001 |
| < 30 | 190 (16.46) | 52 (62.65) | 63 (73.26) | 17 (44.74) | 299 (72.75) |  |  |
| 30-49 | 107 (7.28) | 23 (27.71) | 22 (25.58) | 20 (52.63) | 95 (23.11) |  |  |
| ≥ 50 | 19 (2.53) | 8 (9.64) | 1 (1.16) | 1 (2.63) | 17 (4.14) |  |  |
| Marital status |  |  |  |  |  | 22.94 | 0.003 |
| Married | 243 (20.25) | 64 (77.11) | 77 (89.53) | 29 (76.32) | 362 (88.08) |  |  |
| Unmarried | 53 (3.8) | 12 (14.46) | 7 (8.14) | 6 (15.79) | 36 (8.76) |  |  |
| Divorced/widowed | 20 (2.22) | 7 (8.43) | 2 (2.33) | 3 (7.89) | 13 (3.16) |  |  |
| Educational level |  |  |  |  |  | 19.36 | 0.013 |
| College and above | 187 (15.51) | 49 (59.04) | 66 (76.74) | 19 (50) | 262 (63.75) |  |  |
| High school or technical school | 69 (6.33) | 20 (24.1) | 14 (16.28) | 9 (23.68) | 99 (24.09) |  |  |
| Junior high school and below | 60 (4.43) | 14 (16.87) | 6 (6.98) | 10 (26.32) | 50 (12.17) |  |  |
| Ethnicity |  |  |  |  |  | 9.22 | 0.324 |
| Han | 185 (13.92) | 44 (53.01) | 46 (53.49) | 22 (57.89) | 256 (62.29) |  |  |
| Zhuang | 113 (11.71) | 37 (44.58) | 36 (41.86) | 12 (31.58) | 135 (32.85) |  |  |
| Other | 18 (0.63) | 2 (2.41) | 4 (4.65) | 4 (10.53) | 20 (4.87) |  |  |
| Occupation |  |  |  |  |  | 18.95 | 0.525 |
| Unemployed | 80 (7.91) | 25 (30.12) | 30 (34.88) | 9 (23.68) | 96 (23.36) |  |  |
| Individual business | 52 (3.48) | 11 (13.25) | 8 (9.3) | 6 (15.79) | 62 (15.09) |  |  |
| Clerk | 68 (5.7) | 18 (21.69) | 18 (20.93) | 8 (21.05) | 97 (23.6) |  |  |
| Student | 54 (3.16) | 10 (12.05) | 19 (22.09) | 5 (13.16) | 83 (20.19) |  |  |
| Services | 27 (2.22) | 7 (8.43) | 4 (4.65) | 3 (7.89) | 38 (9.25) |  |  |
| Workers | 35 (3.8) | 12 (14.46) | 7 (8.14) | 7 (18.42) | 35 (8.52) |  |  |
| Region |  |  |  |  |  | 12.86 | 0.683 |
| Xixiangtang | 81 (8.23) | 26 (31.33) | 28 (32.56) | 11 (28.95) | 136 (33.09) |  |  |
| Qingxiu | 80 (5.7) | 18 (21.69) | 22 (25.58) | 11 (28.95) | 103 (25.06) |  |  |
| Jiangnan | 48 (3.8) | 12 (14.46) | 14 (16.28) | 5 (13.16) | 53 (12.9) |  |  |
| Xingning | 30 (3.8) | 12 (14.46) | 7 (8.14) | 4 (10.53) | 28 (6.81) |  |  |
| Other | 77 (4.75) | 15 (18.07) | 15 (17.44) | 7 (18.42) | 91 (22.14) |  |  |
| Infection of other STDs |  |  |  |  |  | 2.45 | 0.653 |
| Yes | 62 (5.38) | 17 (20.48) | 14 (16.28) | 5 (13.16) | 67 (16.3) |  |  |
| No | 254 (20.89) | 66 (79.52) | 72 (83.72) | 33 (86.84) | 344 (83.7) |  |  |
| Number of sexual partners |  |  |  |  |  | 1.48 | 0.993 |
| 1-4 | 112 (9.49) | 30 (36.14) | 28 (32.56) | 13 (34.21) | 143 (34.79) |  |  |
| 5-9 | 90 (6.33) | 20 (24.1) | 26 (30.23) | 12 (31.58) | 113 (27.49) |  |  |
| ≥10 | 113 (10.44) | 33 (39.76) | 32 (37.21) | 13 (34.21) | 155 (37.71) |  |  |
| Infection time |  |  |  |  |  | 6.57 | 0.583 |
| Recent | 101 (8.23) | 26 (31.33) | 35 (40.7) | 15 (39.47) | 136 (33.09) |  |  |
| Long-term | 187 (14.56) | 46 (55.42) | 42 (48.84) | 18 (47.37) | 240 (58.39) |  |  |
| Unknow | 28 (3.48) | 11 (13.25) | 9 (10.47) | 5 (13.16) | 35 (8.52) |  |  |
| Subtype |  |  |  |  |  | 500.03 | <0.001 |
| CRF01_AE | 122 (10.13) | 32 (38.55) | 53 (61.63) | 38 (100) | 0 (0) |  |  |
| CRF07_BC | 120 (3.8) | 12 (14.46) | 24 (27.91) | 0 (0) | 340 (82.73) |  |  |
| CRF55_01B | 26 (7.59) | 24 (28.92) | 0 (0) | 0 (0) | 71 (17.27) |  |  |
| Other | 48 (4.75) | 15 (18.07) | 9 (10.47) | 0 (0) | 0 (0) |  |  |

Table S2 Characteristics analysis of different degree.

| Variables | Degree in network（%） | | | | *χ ^2^* | *p* |
| --- | --- | --- | --- | --- | --- | --- |
|  | 0 | 1 | 2-3 | ≥4 |  |  |
| Age |  |  |  |  | 19.59 | 0.003 |
| < 30 | 190 (30.53) | 58 (60.42) | 65 (62.5) | 308 (73.68) |  |  |
| 30-49 | 107 (16.84) | 32 (33.33) | 31 (29.81) | 97 (23.21) |  |  |
| ≥ 50 | 19 (3.16) | 6 (6.25) | 8 (7.69) | 13 (3.11) |  |  |
| Marital status |  |  |  |  | 460.6 | <0.001 |
| Married | 53 (38.95) | 74 (77.08) | 84 (80.77) | 374 (89.47) |  |  |
| Unmarried | 243 (8.95) | 17 (17.71) | 12 (11.54) | 32 (7.66) |  |  |
| Divorced/widowed | 20 (2.63) | 5 (5.21) | 8 (7.69) | 12 (2.87) |  |  |
| Educational level |  |  |  |  | 11.56 | 0.073 |
| College and above | 187 (34.21) | 65 (67.71) | 60 (57.69) | 273 (65.31) |  |  |
| High school or technical school | 69 (10) | 19 (19.79) | 24 (23.08) | 99 (23.68) |  |  |
| Junior high school and below | 60 (7.37) | 14 (14.58) | 20 (19.23) | 46 (11) |  |  |
| Ethnicity |  |  |  |  | 1.61 | 0.952 |
| Han | 185 (29.47) | 56 (58.33) | 67 (64.42) | 245 (58.61) |  |  |
| Zhuang | 113 (18.42) | 35 (36.46) | 33 (31.73) | 152 (36.36) |  |  |
| Other | 18 (2.63) | 5 (5.21) | 4 (3.85) | 21 (5.02) |  |  |
| Occupation |  |  |  |  | 13.64 | 0.553 |
| Unemployed | 80 (13.16) | 25 (26.04) | 27 (25.96) | 108 (25.84) |  |  |
| Individual business | 52 (7.37) | 14 (14.58) | 17 (16.35) | 56 (13.4) |  |  |
| Clerk | 68 (15.26) | 29 (30.21) | 20 (19.23) | 92 (22.01) |  |  |
| Student | 54 (4.74) | 9 (9.38) | 18 (17.31) | 90 (21.53) |  |  |
| Services | 27 (4.74) | 9 (9.38) | 8 (7.69) | 35 (8.37) |  |  |
| Workers | 35 (5.26) | 10 (10.42) | 14 (13.46) | 37 (8.85) |  |  |
| Region |  |  |  |  | 16.01 | 0.191 |
| Xixiangtang | 81 (14.21) | 27 (28.13) | 40 (38.46) | 134 (32.06) |  |  |
| Qingxiu | 80 (10) | 19 (19.79) | 21 (20.19) | 114 (27.27) |  |  |
| Jiangnan | 48 (8.42) | 16 (16.67) | 12 (11.54) | 56 (13.4) |  |  |
| Xingning | 30 (6.32) | 12 (12.5) | 5 (4.81) | 34 (8.13) |  |  |
| Other | 77 (10.53) | 20 (20.83) | 26 (25) | 80 (19.14) |  |  |
| Infection of other STDs |  |  |  |  | 1.4 | 0.705 |
| Yes | 62 (8.95) | 17 (17.71) | 18 (17.31) | 68 (16.27) |  |  |
| No | 254 (41.58) | 79 (82.29) | 86 (82.69) | 350 (83.73) |  |  |
| Number of sexual partners |  |  |  |  | 2.39 | 0.882 |
| 1-4 | 112 (18.95) | 36 (37.5) | 36 (34.62) | 142 (33.97) |  |  |
| 5-9 | 90 (13.68) | 26 (27.08) | 24 (23.08) | 121 (28.95) |  |  |
| ≥10 | 113 (17.89) | 34 (35.42) | 44 (42.31) | 155 (37.08) |  |  |
| Infection time |  |  |  |  | 6.62 | 0.357 |
| Recent | 101 (18.95) | 36 (37.5) | 30 (28.85) | 146 (34.93) |  |  |
| Long-term | 187 (24.74) | 47 (48.96) | 61 (58.65) | 238 (56.94) |  |  |
| Unknow | 28 (6.84) | 13 (13.54) | 13 (12.5) | 34 (8.13) |  |  |
| Subtype |  |  |  |  | 123.65 | <0.001 |
| CRF01_AE | 122 (14.21) | 27 (28.13) | 21 (20.19) | 75 (17.94) |  |  |
| CRF07_BC | 120 (20) | 38 (39.58) | 56 (53.85) | 282 (67.46) |  |  |
| CRF55_01B | 26 (10.53) | 20 (20.83) | 22 (21.15) | 53 (12.68) |  |  |
| Other | 48 (5.79) | 11 (11.46) | 5 (4.81) | 8 (1.91) |  |  |

Table S3 Characteristics of cluster growth rate.

| Variables | Growth rate of HIV-1 transmission cluster (n, %) | | | | P value |
| --- | --- | --- | --- | --- | --- |
|  | Low (n=447) | Medium (n=236) | High (n=207) | Total (n=890) |  |
| Age |  |  |  |  | 0.130 |
| < 30 | 282 (0.63) | 170 (0.72) | 145 (0.70) | 597 (0.67) |  |
| 30-49 | 141 (0.32) | 57 (0.24) | 51 (0.25) | 249 (0.28) |  |
| ≥ 50 | 24 (0.05) | 9 (0.04) | 11 (0.05) | 44 (0.05) |  |
| Marital status |  |  |  |  | 0.392 |
| Married | 61 (0.14) | 22 (0.09) | 21 (0.10) | 104 (0.12) |  |
| Unmarried | 363 (0.81) | 202 (0.86) | 178 (0.86) | 743 (0.83) |  |
| Divorced/widowed | 23 (0.05) | 12 (0.05) | 8 (0.04) | 43 (0.05) |  |
| Educational level |  |  |  |  | 0.166 |
| College and above | 280 (0.63) | 157 (0.67) | 126 (0.61) | 563 (0.63) |  |
| High school or technical school | 90 (0.20) | 51 (0.22) | 54 (0.26) | 195 (0.22) |  |
| Junior high school and below | 77 (0.17) | 28 (0.12) | 27 (0.13) | 132 (0.15) |  |
| Ethnicity |  |  |  |  | 0.844 |
| Han | 260 (0.58) | 138 (0.58) | 128 (0.62) | 526 (0.59) |  |
| Zhuang | 167 (0.37) | 86 (0.36) | 68 (0.33) | 321 (0.36) |  |
| Other | 20 (0.04) | 12 (0.05) | 11 (0.05) | 43 (0.05) |  |
| Subtype |  |  |  |  | <0.001 |
| CRF01_AE | 172 (0.38) | 48 (0.20) | 0 (0.00) | 220 (0.25) |  |
| CRF07_BC | 167 (0.37) | 129 (0.55) | 189 (0.91) | 485 (0.54) |  |
| CRF55_01B | 50 (0.11) | 52 (0.22) | 18 (0.09) | 120 (0.13) |  |
| Other | 58 (0.13) | 7 (0.03) | 0 (0.00) | 65 (0.07) |  |
| Occupation |  |  |  |  | 0.261 |
| Unemployed | 120 (0.27) | 60 (0.25) | 49 (0.24) | 229 (0.26) |  |
| Individual business | 67 (0.15) | 30 (0.13) | 37 (0.18) | 134 (0.15) |  |
| Student | 81 (0.18) | 36 (0.15) | 47 (0.23) | 164 (0.18) |  |
| Clerk | 99 (0.22) | 62 (0.26) | 38 (0.18) | 199 (0.22) |  |
| Workers | 48 (0.11) | 29 (0.12) | 16 (0.08) | 93 (0.10) |  |
| Services | 32 (0.07) | 19 (0.08) | 20 (0.10) | 71 (0.08) |  |
| Infection of other STDs |  |  |  |  | 0.410 |
| No | 362 (0.81) | 197 (0.83) | 176 (0.85) | 735 (0.83) |  |
| Yes | 85 (0.19) | 39 (0.17) | 31 (0.15) | 155 (0.17) |  |
| Number of sexual partners |  |  |  |  | 0.134 |
| 1-4 | 161 (0.36) | 66 (0.28) | 80 (0.39) | 307 (0.34) |  |
| 5-9 | 124 (0.28) | 71 (0.30) | 50 (0.24) | 245 (0.28) |  |
| ≥10 | 162 (0.36) | 99 (0.42) | 77 (0.37) | 338 (0.38) |  |
| Infection time |  |  |  |  | <0.001 |
| Long-term | 253 (0.57) | 114 (0.48) | 132 (0.64) | 499 (0.56) |  |
| Recent | 151 (0.34) | 105 (0.44) | 49 (0.24) | 305 (0.34) |  |
| Unknow | 43 (0.10) | 17 (0.07) | 26 (0.13) | 86 (0.10) |  |
| CD4 count |  |  |  |  | 0.024 |
| < 200 | 69 (0.15) | 37 (0.16) | 19 (0.09) | 125 (0.14) |  |
| 200-350 | 136 (0.30) | 92 (0.39) | 70 (0.34) | 298 (0.33) |  |
| ﹥350 | 242 (0.54) | 107 (0.45) | 118 (0.57) | 467 (0.52) |  |
| Viral load (Log10) |  |  |  |  | <0.001 |
| <4 | 32 (0.07) | 21 (0.09) | 11 (0.05) | 64 (0.07) |  |
| 4-5 | 129 (0.29) | 92 (0.39) | 28 (0.14) | 249 (0.28) |  |
| ≥5 | 77 (0.17) | 73 (0.31) | 22 (0.11) | 172 (0.19) |  |
| Unknow | 209 (0.47) | 50 (0.21) | 146 (0.71) | 405 (0.46) |  |
| Region |  |  |  |  | 0.216 |
| Xixiangtang | 121 (0.27) | 86 (0.36) | 67 (0.32) | 274 (0.31) |  |
| Qingxiu | 105 (0.23) | 62 (0.26) | 52 (0.25) | 219 (0.25) |  |
| Jiangnan | 68 (0.15) | 32 (0.14) | 27 (0.13) | 127 (0.14) |  |
| Xingning | 43 (0.10) | 18 (0.08) | 15 (0.07) | 76 (0.09) |  |
| Liangqing | 22 (0.05) | 10 (0.04) | 12 (0.06) | 44 (0.05) |  |
| Other | 88 (0.20) | 28 (0.12) | 34 (0.16) | 150 (0.17) |  |
| Year of diagnosis* |  |  |  |  | <0.001 |
| 2016 | 65 (0.15) | (0.00) | (0.00) | 65 (0.07) |  |
| 2017 | 66 (0.15) | 17 (0.07) | 75 (0.36) | 158 (0.18) |  |
| 2018 | 71 (0.16) | 33 (0.14) | 70 (0.34) | 174 (0.20) |  |
| 2019 | 83 (0.19) | 28 (0.12) | 62 (0.30) | 173 (0.19) |  |
| 2020 | 87 (0.19) | 72 (0.31) | 0 (0.00) | 159 (0.18) |  |
| 2021 | 75 (0.17) | 86 (0.36) | 0 (0.00) | 161 (0.18) |  |

*Fisher's Exact Test was used for the statistical analysis.
